# Supplementary material for: A digital 3D reference atlas reveals cellular growth patterns shaping the Arabidopsis ovule
Source: eLife. 2021 Jan 6;10:e63262. doi: 10.7554/eLife.63262 (PMC7787667; doi:10.7554/eLife.63262)
Supplement: Supplementary file 1. [file elife-63262-supp1.docx]

**Supplementary File 1. Standardized cell type labels**

| **Cell type** | **Morphological definition of cell type** | **Standardized cell type label** |
| --- | --- | --- |
| **Organization** |  |  |
| L1 | Outermost cell layer (epidermis) | L1 |
| L2 | First sub-epidermal cell layer | L2 |
| L3 | All interior cells enclosed by the L2 layer | L3 |
| Distal |  | D |
| Proximal |  | P |
| Anterior | Orientation of ovule relative to gynoecium, ovule tissue oriented towards the stigma (tip) | ANT |
| Posterior | Orientation of ovule relative to gynoecium, ovule tissue oriented towards the gynophore (bottom) | POST |
| Lateral |  | LAT |
| Median |  | MED |
| Adaxial (dorsal) |  | AD |
| Abaxial (ventral) |  | AB |
|  |  |  |
| **Tissues along proximal-distal axis** |  |  |
| Nucellus | Distal region, harbors the mmc, proximally delineated by adaxial inner integument | n |
| Chalaza | Central region, flanked by the two integuments, distal end marked by adaxial inner integument, proximal end marked by abaxial outer integument, does not include epidermis | c |
| Distal chalaza | Formed by the chalazal cells underlying the inner integument, region is misspecified or missing in *wus* mutants^a^ | dc |
| Proximal chalaza | Formed by the chalazal cells underlying the outer integument | pc |
| Anterior chalaza | Group of proximal chalazal cells, positioned at the anterior side, underlying the epidermal cells forming the frontal base of the hood-like structure generated by the epidermis-derived outer integument | ac |
| Posterior chalaza | Group of proximal chalazal cells, positioned opposite to the cells of the anterior chalaza | pc |
| Funiculus | Proximal region, stalk-like structure, carries the vascular strand, distal end marked by chalaza, proximal end marked by placenta | f |
|  |  |  |
| **Integuments** |  |  |
| Abaxial outer integument | Outermost (ventral) single cell layer of outer integument, entirely made of epidermal cells, proximal end marked by chalaza | Aboi (oi2) |
| Adaxial outer integument | Innermost (dorsal) single cell layer of outer integument, proximal end marked by chalaza | Adoi (oi1) |
| Abaxial inner integument | Outer (ventral) single cell layer of inner integument, entirely made of epidermal cells, proximal end marked by chalaza | Abii (ii2) |
| Adaxial inner integument | Inner (dorsal) single cell layer of inner integument, entirely made of epidermal cells, proximal end marked by chalaza | Adii (ii1) |
| Parenchymatic extra layer in late inner integument (Schneitz layer) | Cell layer derived from adaxial inner integument, proximal end marked by chalaza | ii1’ |
| Tip cell | Small cell at the micropylar (distal) rim of each of the two developing integuments | tc |
|  |  |  |
| **Megasporogenesis** |  |  |
| MMC | Single large L2 cell in distal end of nucellus, eventually undergoing meiosis, volume ≥ 335 µm^3^ | mmc |
| Tetrad | Product of meiosis undergone by mmc, multi-planar or linear tetrad of haploid megaspores | tet |
|  |  |  |
| **Megagametogenesis^b^** |  |  |
| Embryo sac | Haploid female gametophyte, encompasses all stages up to but not including fertilization | es |
| Embryo sac (FG1) | Mono-nuclear, tear-drop-shaped cell | fg1 |
| Embryo sac (FG2) | Early two-nucleate | fg2 |
| Embryo sac (FG3) | Late two-nucleate, large vacuole prominent | fg3 |
| Embryo sac (FG4) | Four-nucleate, two nuclei at each pole | fg4 |
| Embryo sac (FG5) | Eight-nucleate, seven celled, polar nuclei unfused | fg5 |
| Embryo sac (FG6) | Seven-celled, polar nuclei fused | fg6 |
| Embryo sac (FG7) | Four-celled, three antipodal cells have degenerated | fg7 |
| Embryo sac (FG8) | Three-celled, one synergid has degenerated | fg8 |
| Egg cell |  | ec |
| Central cell |  | cc |
| Antipodal cell |  | ap |
|  |  |  |
| **Cell cycle** |  |  |
| M-phase | Mitotic figures (metaphase, anaphase) | M |
| Interphase | Cells in G0, S, or G2 phase | I |

^a^As described in [(Sieber et al. 2004)](http://sciwheel.com/work/citation?ids=1643183&pre=&suf=&sa=0).

^b^Stages according to [(Christensen et al. 1997)](http://f1000.com/work/citation?ids=7253956&pre=&suf=&sa=0).
