# Supplementary material for: A digital 3D reference atlas reveals cellular growth patterns shaping the Arabidopsis ovule
Source: eLife. 2021 Jan 6;10:e63262. doi: 10.7554/eLife.63262 (PMC7787667; doi:10.7554/eLife.63262)
Supplement: Supplementary file 2. [file elife-63262-supp2.docx]

**Supplementary File 2. Pistil length of individual live pistils monitored up to 192 hours**

|  | Pistil Nr | | | | | |  |  |
| --- | --- | --- | --- | --- | --- | --- | --- | --- |
| Hours | 1^a^ | 2^a^ | 3^a^ | 4^a^ | 5^a^ | 6^a^ | Mean | SD |
| 0 | 0,31 | 0,32 |  |  |  |  | 0,315 | 0,007 |
| 24 | 0,54 | 0,49 | 0,45 | 0,47 |  |  | 0,488 | 0,039 |
| 48 | 0,74 | 0,78 | 0,66 | 0,64 | 0,77 | 0,63 | 0,703 | 0,068 |
| 72 | 0,97 | 1 | 1 | 1 | 1 |  | 0,994 | 0,013 |
| 96 | 1,31 | 1,33 | 1,29 | 1,22 | 1,38 |  | 1,306 | 0,059 |
| 120 | 1,81 | 1,8 | 1,85 | 1,73 | 1,73 |  | 1,784 | 0,053 |
| 144 | 2,3 | 2,28 | 2,54 | 2,15 | 2,3 |  | 2,314 | 0,141 |
| 168 | 2,75 | 3,26 | 3 |  |  |  | 3,003 | 0,255 |
| 192 | 3,7 | 4 | 4 |  |  |  | 3,900 | 0,173 |

^a^Pistil length in mm

**Curve fitting**

Model: y = y0 + a(-1 + exp(x/b))

y0 = pistil length at 0 hours (0.32)

a = fitting parameter (0.66)

b = fitting parameter (103.1)

x = time

**Goodness of Fit**

Degrees of Freedom: 35
R^2^: 0.9918

Sum of Squares: 0.3282

Sy.x: 0.09683
