# Supplementary material for: A digital 3D reference atlas reveals cellular growth patterns shaping the Arabidopsis ovule
Source: eLife. 2021 Jan 6;10:e63262. doi: 10.7554/eLife.63262 (PMC7787667; doi:10.7554/eLife.63262)
Supplement: Supplementary file 3. [file elife-63262-supp3.docx]

**Supplementary File 3. Correlation of pistil length with ovule stages**

| Pistil length (mm) | Number of ovules counted | Ovule stage | | | | | | | | | | | | |
| --- | --- | --- | --- | --- | --- | --- | --- | --- | --- | --- | --- | --- | --- | --- |
|  | | 1-I | 1-II | 2-I | 2-II | 2-III | 2-IV | 2-V | 3-I | 3-II | 3-III | 3-IV | 3-V | 3-VI |
| 0.33 | 17 | 17 | - | - | - | - | - | - | - | - | - | - | - | - |
| 0.38 | 21 | 21 | - | - | - | - | - | - | - | - | - | - | - | - |
| 0.38 | 13 | 13 | - | - | - | - | - | - | - | - | - | - | - | - |
| 0.44 | 25 | 12 | 13 | - | - | - | - | - | - | - | - | - | - | - |
| 0.45 | 11 | 11 | 12 | - | - | - | - | - | - | - | - | - | - | - |
| 0.49 | 24 | 7 | 13 | 4 | - | - | - | - | - | - | - | - | - | - |
| 0.51 | 27 | 8 | 19 | - | - | - | - | - | - | - | - | - | - | - |
| 0.57 | 24 | - | 2 | 22 | - | - | - | - | - | - | - | - | - | - |
| 0.61 | 25 | - | 10 | 15 | - | - | - | - | - | - | - | - | - | - |
| 0.62 | 16 | 3 | 6 | 7 | - | - | - | - | - | - | - | - | - | - |
| 0.62 | 16 | - | 8 | 8 | - | - | - | - | - | - | - | - | - | - |
| 0.66 | 18 | - | 1 | 8 | 9 | - | - | - | - | - | - | - | - | - |
| 0.68 | 20 | - | 1 | 8 | 11 | - | - | - | - | - | - | - | - | - |
| 0.69 | 22 | - | - | 9 | 13 | - | - | - | - | - | - | - | - | - |
| 0.71 | 25 | - | - | 10 | 13 | - | - | - | - | - | - | - | - | - |
| 0.72 | 27 | - | - | 7 | 16 | 4 | - | - | - | - | - | - | - | - |
| 0.75 | 29 | - | - | 2 | 13 | 14 | - | - | - | - | - | - | - | - |
| 0.82 | 41 | - | - | 8 | 22 | 11 | - | - | - | - | - | - | - | - |
| 0.83 | 21 | - | - | - | 10 | 11 | - | - | - | - | - | - | - | - |
| 0.88 | 18 | - | - | - | - | 15 | 3 | - | - | - | - | - | - | - |
| 0.94 | 20 | - | - | - | - | 6 | 10 | 4 | - | - | - | - | - | - |
| 0.95 | 31 | - | - | - | 5 | 10 | 13 | 3 | - | - | - | - | - | - |
| 0.95 | 26 | - | - | - | 1 | 16 | 13 | - | - | - | - | - | - | - |
| 0.98 | 29 | - | - | - | - | 13 | 7 | 8 | 1 | - | - | - | - | - |
| 1.1 | 27 | - | - | - | - | 6 | 8 | 13 |  | - | - | - | - | - |
| 1.13 | 25 | - | - | - | - | 2 | 5 | 9 | 9 | - | - | - | - | - |
| 1.15 | 28 | - | - | - | 1 | 2 | 7 | 12 | 6 | - | - | - | - | - |
| 1.18 | 20 | - | - | - | - | 4 | 10 | 6 | - | - | - | - | - | - |
| 1.2 | 43 | - | - | - | - | 3 | 13 | 19 | 8 | - | - | - | - | - |
| 1.2 | 34 | - | - | - | - | 4 | 10 | 16 | 4 | - | - | - | - | - |
| 1.2 | 33 | - | - | - | - | - | - | 12 | 20 | 1 | - | - | - | - |
| 1.21 | 24 | - | - | - | - | 3 | 8 | 13 | - | - | - | - | - | - |
| 1.22 | 32 | - | - | - | - | - | - | 6 | 13 | 13 | - | - | - | - |
| 1.28 | 22 | - | - | - | - | 3 | 5 | 10 | 4 | - | - | - | - | - |
| 1.3 | 24 | - | - | - | - | - | - | - | 19 | 4 | 1 | - | - | - |
| 1.31 | 18 | - | - | - | - | - | - | - | 13 | 4 | 1 | - | - | - |
| 1.32 | 25 | - | - | - | - | - | 2 | 8 | 15 | - | - | - | - | - |
| 1.35 | 28 | - | - | - | - | - | - | 6 | 22 | - | - | - | - | - |
| 1.46 | 19 | - | - | - | - | - | - | 7 | 12 | - | - | - | - | - |
| 1.49 | 36 | - | - | - | - | - | - | 1 | 19 | 9 | 6 | 1 | - | - |
| 1.55 | 41 | - | - | - | - | - | - | - | 15 | 10 | 11 | 5 | - | - |
| 1.55 | 20 | - | - | - | - | - | - | - | 13 | 4 | 3 | - | - | - |
| 1.6 | 30 | - | - | - | - | - | - | 1 | 3 | 12 | 10 | 4 | - | - |
| 1.61 | 24 | - | - | - | - | - | - | 2 | 6 | 9 | 7 | - | - | - |
| 1.62 | 24 | - | - | - | - | - | - | - | 4 | 6 | 7 | 5 | 2 | - |
| 1.63 | 25 | - | - | - | - | - | - | - | - | 2 | 7 | 11 | 5 | - |
| 1.65 | 23 | - | - | - | - | - | - | - | 2 | 8 | 11 | 2 | - | - |
| 1.71 | 19 | - | - | - | - | - | - | - | 1 | 1 | 1 | 12 | 4 | - |
| 1.72 | 25 | - | - | - | - | - | - | - | - | 1 | - | 11 | 12 | 1 |
| 1.75 | 25 | - | - | - | - | - | - | - | - | - | 2 | 7 | 10 | 6 |
| 1.79 | 37 | - | - | - | - | - | - | - | - | - | 3 | 3 | 21 | 8 |
| 1.82 | 28 | - | - | - | - | - | - | - | 3 | 4 | 5 | 12 | 4 | - |
| 1.84 | 29 | - | - | - | - | - | - | - | - | - | 2 | 14 | 13 | - |
| 1.9 | 21 | - | - | - | - | - | - | - | - | 2 | 3 | 10 | 6 | - |
| 1.96 | 17 | - | - | - | - | - | - | - | - | - | - | 5 | 12 | 5 |
| 1.97 | 19 | - | - | - | - | - | - | - | - | - | 4 | 2 | 13 | - |
| 2.1 | 23 | - | - | - | - | - | - | - | - | - | - | - | 17 | 6 |
| 2.1 | 14 | - | - | - | - | - | - | - | - | - | - | - | 12 | 2 |
| 2.1 | 27 | - | - | - | - | - | - | - | - | 5 | 3 | 7 | 12 | - |
| 2.18 | 18 | - | - | - | - | - | - | - | - | - | - | 15 | 13 | - |
| 2.2 | 28 | - | - | - | - | - | - | - | - | 2 | - | 11 | 10 | 5 |
| 2.27 | 18 | - | - | - | - | - | - | - | - | - | - | - | 2 | 16 |
| 2.3 | 20 | - | - | - | - | - | - | - | - | - | - | - | 7 | 13 |
| 2.32 | 14 | - | - | - | - | - | - | - | - | - | - | - | - | 14 |
