# Supplementary material for: A digital 3D reference atlas reveals cellular growth patterns shaping the Arabidopsis ovule
Source: eLife. 2021 Jan 6;10:e63262. doi: 10.7554/eLife.63262 (PMC7787667; doi:10.7554/eLife.63262)
Supplement: Supplementary file 4. [file elife-63262-supp4.docx]

**Supplementary File 4. Cell numbers and total volumes of *ino-5* ovules staged according to the wild-type cohort with removed outer integument.**

| Stage^a,b^ | N Cells wild type (-oi) | Volume (x10^4^ μm^3^) wild type (-oi) | N Cells *ino-5* | Volume (x10^4^ μm^3^) *ino-5* |
| --- | --- | --- | --- | --- |
| 1-II | 74.0 ± 17.1 | 1.0 ± 0.2 | 87 ± 23.1 | 1.1 ± 0.3 |
| 2-I | 176.9 ± 31.5 | 2.5 ± 0.4 | 166.2 ± 19.5 | 2.1 ± 0.1 |
| 2-II | 220.6 ± 24.9 | 2.7 ± 0.6 | 241.1 ± 9.1 | 3.0 ± 0.2 |
| 2-III | 270.3 ± 32.4 | 3.2 ± 0.6 | 305.5 ± 34.6 | 3.7 ± 0.3 |
| 2-IV | 380.5 ± 26.5 | 4.5 ± 0.4 | 382.3 ± 45.7 | 4.1 ± 0.5 |
| 2-V | 524.7 ± 66.3 | 6.2 ± 1.0 | 536.7 ± 60.8 | 6.4 ± 0.9 |
| 3-I | 712.2 ± 61.2 | 9.2 ± 1.1 | 716.9 ± 58.8 | 9.4 ± 0.9 |
| 3-II | 888.4 ± 52.9 | 13.8 ± 1.3 | 858.0 ± 28.7 | 12.7 ± 0.9 |
| 3-III | 951.2 ± 90.1 | 14.9 ± 2.1 | 956.7 ± 28.9 | 15.5 ± 0.6 |
| 3-IV | 1037 ± 103.7 | 18.1 ± 2.2 | 1102 ± 49.5 | 19.6 ± 2.1 |
| 3-V | 1140 ± 96.8 | 20.8 ± 2.3 | 1241 ± 32.2 | 23.5 ± 17.7 |
| 3-VI | 1345 ± 131.9 | 26.6 ± 3.7 | 1383 ± 61.1 | 25.7 ± 16.8 |

^a^Number of 3D digital ovules scored: 10 (stages 2-II- 3-II, 3-IV, 3-VI), 11 (stages 2-I, 3-III, 3-V), 13 (stage 2-II), 14 (stage 1-I), 28 (stage 1-II).

Values represent mean ± SD.

^b^Number of 3D digital ovules scored for *ino-5* dataset: 3 (stages 2-IV), 6 (stages 2-III), 7 (stages 2-II, 3-III, 3-VI), 9 (stages 3-II), 10 (stages 2-I, 3-V), 12 (stages 1-II), 14 (stages 3-I, 3-IV), 20 (stages 2-V).

Values represent mean ± SD.
